# CRISPR-HOLMES-based NAD^+^ detection

**DOI:** 10.3389/fbioe.2024.1355640

**Published:** 2024-03-25

**Authors:** Songkuan Zhuang, Tianshuai Hu, Hongzhong Zhou, Shiping He, Jie Li, Yuehui Zhang, Dayong Gu, Yong Xu, Yijian Chen, Jin Wang

**Affiliations:** ^1^ Guangdong Key Laboratory for Biomedical Measurements and Ultrasound Imaging, National-Regional Key Technology Engineering Laboratory for Medical Ultrasound, School of Biomedical Engineering, Shenzhen University Medical School, Shenzhen, China; ^2^ Department of Clinical Laboratory, Shenzhen Institute of Translational Medicine, The First Affiliated Hospital of Shenzhen University, Shenzhen Second People’s Hospital, Shenzhen, China; ^3^ Shenzhen Bao An Peoples Hospital, Shenzhen, China; ^4^ Department of Clinical Laboratory, Shenzhen Third People’s Hospital, The Second Affiliated Hospital, School of Medicine, Southern University of Science and Technology, National Clinical Research Center for Infectious Disease, Shenzhen, China; ^5^ Institute of Antibiotics, Huashan Hospital, Fudan University & Key Laboratory of Clinical Pharmacology of Antibiotics, National Health Commission, Shanghai, China; ^6^ Shanghai Tolo Biotechnology Co Ltd, Shanghai, China

**Keywords:** CRISPR, Cas12a, HOLMES, NAD^+^, acetylation

## Abstract

Studies have indicated that the intracellular nicotinamide adenine dinucleotide (NAD^+^) level is associated with the occurrence and development of many diseases. However, traditional nicotinamide adenine dinucleotide (NAD^+^) detection techniques are time-consuming and may require large and expensive instruments. We recently found that the clustered regularly interspaced short palindromic repeat (CRISPR)-Cas12a protein can be inactivated by AcrVA5-mediated acetylation and reactivated by CobB, using NAD^+^ as the co-factor. Therefore, in this study, we created a CRISPR-Cas12a-based one-step HOLMES(NAD^+^) system for rapid and convenient NAD^+^ detection with the employment of both acetylated Cas12a and CobB. In HOLMES(NAD^+^), acetylated Cas12a loses its *trans*-cleavage activities and can be reactivated by CobB in the presence of NAD^+^, cutting ssDNA reporters to generate fluorescence signals. HOLMES(NAD^+^) shows both sensitivity and specificity in NAD^+^ detection and can be used for quantitative determination of intracellular NAD^+^ concentrations. Therefore, HOLMES(NAD^+^) not only provides a convenient and rapid approach for target NAD^+^ quantitation but also expands the application scenarios of HOLMES to non-nucleic acid detection.

## 1 Introduction

Nicotinamide adenine dinucleotide (NAD^+^) is essential for cellular catabolic metabolism, participating as a coenzyme in redox reactions and serving as an important co-substrate for NAD^+^-dependent enzymes including the sirtuin family of protein deacetylases and the PARP family of DNA repair enzymes. As a result, the quantitative detection of the NAD^+^ level may help reflect the status of an organism and preclude the onset and progression of metabolism-related diseases.

So far, a wide variety of technologies have been developed for the measurements of NAD^+^, including high-performance liquid chromatography (HPLC) (Jones, 1981), liquid chromatography/electrospray ionization tandem mass spectrometry (MS) (Yamada et al., 2006), enzymatic assays (Bernofsky and Swan, 1973), and genetically encoded fluorescent biosensor (Yu et al., 2019). However, these techniques are either time-consuming or may require large and expensive instruments, resulting in much inconvenience. For example, the HPLC method has low sensitivity and requires more samples when compared with other techniques. Although the MS method possesses high accuracy and high specificity, it relies on expensive precision instruments and professional operations. The enzymatic method, also known as the colorimetric NAD^+^ assay, needs two steps to calculate the NAD^+^ amount. First, the total amount of NAD^+^ and NADH and the amount of NADH are measured, and then, the NAD^+^ concentration was indirectly obtained by subtraction. Although the bioluminescent method has shown advantages in sensitivity, rapidness, and convenience, its specificity needs further improvement as it may have difficulty in distinguishing low-concentration NAD^+^ from high-concentration NADH samples. Therefore, it is necessary to develop a rapid, reliable, cost-effective, and quantitative method for convenient NAD^+^ detection.

The clustered regularly interspaced short palindromic repeat (CRISPR)/CRISPR-associated (Cas) systems have been found in many bacteria and most archaea and are well-known as an effective defense means for hosts to prevent invasion from a mobile genetic element (MGE), such as bacteriophages (Mojica et al., 2005). Briefly, Cas proteins specifically recognize and cut target DNA under the guidance of guide RNAs, whose findings have greatly promoted precise genome editing (Jinek et al., 2012). Among these Cas proteins, Cas12 and Cas13 possess not only *cis*-cleavage activities but also *trans*-cleavage activities, where these activities have been employed to develop the next-generation CRISPR diagnostic systems, such as Cas12-based HOLMES (an one-Hour low-cost multipurpose highly efficient system) and Cas13-based SHERLOCK (Gootenberg et al., 2017; Li et al., 2018; Linxian et al., 2019). Previous studies showed that Cas effectors, such as Cas12a, could be regulated by the post-translational acetylation (PTM) of a key lysine site by GNAT-family acetyltransferase, namely, AcrVA5, resulting in its loss of the ability to interact with the protospacer adjacent motif (PAM) site and failure in protecting the hosts by cleaving the invading MGEs (Dong et al., 2019; Knott et al., 2019). Recently, our study has demonstrated that Cas12a, inactivated by AcrVA5, could be reactivated by CobB, NAD^+^-dependent sirtuin-type deacetylase, through a process of deacetylation that is dependent on NAD^+^, and reactivated Cas12a recovers its abilities in both target double-stranded DNA (dsDNA) recognition and cleavage *in vitro* and host defense *in vivo* (Kang et al., 2022). Therefore, one could conclude that NAD^+^ plays an important role in the regulation of Cas12a activities, including the *trans*-cleavage activities.

Based on the above findings, we employed this mechanism to develop a Cas12a-based CRISPR diagnostic system, namely, HOLMES(NAD^+^), for convenient NAD^+^ detection. Our results demonstrate the outstanding performance of HOLMES(NAD^+^), characterized by a low-detection limit (22.5 nM), cost-effectiveness, high specificity, and remarkable repeatability (RSD = 3.80–5.82%) and precision (RSD = 1.65–8.72%). More importantly, HOLMES(NAD^+^) can quantitatively detect NAD^+^ levels in real biological samples within 30 min, following a one-pot and one-step procedure.

## 2 Materials and methods

### 2.1 Construction of plasmids

To construct the LbCas12a expression plasmid pCDF-PylT-LbCas12a, the *LbCas12a* gene was amplified from plasmid pET28a-LbCas12a (ToloBio) with the primers of LbCas12a-F/R with high-fidelity DNA polymerase, and the amplicons were purified and digested with *BamHI* and *SalI* before being inserted into the same sites in pCDF-PylT (Neumann et al., 2009) *via* T4 DNA ligation.

### 2.2 Purification of proteins

The purification procedures of the recombinant proteins were the same, as previously described (Kang et al., 2022). Briefly, to obtain acetylated LbCas12a, the plasmids of pCDF-PylT-LbCas12a and pET28a-AcrVA5 (Dong et al., 2019) were co-transformed into *Escherichia coli* BL21 (DE3), and the transformants were induced by the addition of IPTG at a final concentration of 0.2 mM. Acetylated LbCas12a proteins were purified following the steps of the Ni-NTA column (HisTap^TM^ HP, GE Healthcare), ion exchange chromatography (HiTrap^TM^ SP HP, GE Healthcare), and molecular sieve (HiLoad^TM^ 16/600, Superdex^TM^ 200 pg, GE Healthcare). Proteins were quantitated using the Bradford method and stored at −80 °C in the storage buffer containing 50 mM Tris-HCl, 600 mM NaCl, 2 mM DTT, 0.2 mM EDTA, and 50% glycerol. The purification of CobB was the same as what was described previously (Kang et al., 2022).

### 2.3 Generation of the dsDNA target

To create short dsDNA, two complementary oligonucleotides of both forward and reverse were synthesized (Supplementary Table S2) (Sangon, China) and annealed at a ratio of 5:1 in 1× Taq buffer to form dsDNA, which was then stored at −20°C. The generated dsDNA was diluted to 100 nM with nuclease-free water before use.

To generate FAM-labeled double-stranded DNA targets, the target DNA fragment was amplified from plasmid pClone007s-COL1A2 (Linxian et al., 2019) with the primers of M13-F and M13-R-FAM with a high-fidelity DNA polymerase. Then, purified amplicons were quantitated with the NanoDrop 2000 instrument (Thermo Fisher Scientific) and stored at −20 °C.

### 2.4 CobB-mediated deacetylation of acLbCas12a

To perform the deacetylation reaction, 1 μM acLbCas12a was treated with 1 μM CobB with the addition of 1 mM NAD^+^ in 1× NEB buffer 3.1 in a 20-μL reaction system. The reaction was carried out at 37 °C for 60 min, and the acetylation status of acLbCas12a was then analyzed by Western blot.

### 2.5 *cis-* and *trans-*cleavage assays

For the *in vitro cis*-cleavage assay, CobB-treated acLbCas12a was incubated in a 20-μL reaction system containing 150-ng target dsDNA, 1 μM acetylated LbCas12a, 1 μM CobB, 1 μM crRNA, and 1 mM NAD^+^. The reaction was performed at 37°C for 60 min in 1× NEB buffer 3.1 and then stopped by heating at 80°C for 15 min, followed by immediately chilling on ice before being further analyzed by PAGE.

For the *in vitro trans*-cleavage assay, CobB-treated acLbCas12a was incubated in a 20-μL reaction system containing 0.5 μM acetylated LbCas12a, 1 μM CobB, 0.5 nM target dsDNA, 12.5 nM crRNA, 0.5 μM 8C-FQ-reporter, and 0.5 μM NAD^+^. The reaction was performed at 37 °C in 1× NEB buffer 3.1, and the results were detected in a real-time PCR machine (Applied Biosystems QuantStudio 3) for 30 min with the fluorescence signal collected every 15 s (λex: 488 nm and λem: 535 nm).

### 2.6 Measurement of the cellular NAD^+^ concentration

#### 2.6.1 Extraction of cellular NAD^+^

To measure NAD^+^ levels in cancer cells, cells were first digested with 0.25% trypsin-EDTA (Gibco), rinsed twice with pre-cooled 1× PBS (HyClone) and then harvested by centrifugation at 5,000 rpm for 5 min at 4°C. Meanwhile, cell numbers were determined by Countess 3 (Thermo Fisher). Cell pellets were resuspended in 300–600 μL acidic extraction buffer (0.3 M HCl), according to the cell numbers, incubated at 95°C for 5 min and then cooled immediately on ice, followed by centrifugation at 12,000 rpm for 10 min at 4°C. The acidic extraction solution (200 μL) containing NAD^+^ was neutralized using 180–200 μL alkaline buffer (0.3 M KOH) before being stored at −80°C for the following analysis.

#### 2.6.2 Detection of NAD^+^

To detect NAD^+^, 1–2 μL extraction solution containing NAD^+^ was mixed with the *trans*-cleavage system containing 0.5 μM acetylated LbCas12a, 1 μM CobB, 0.5 nM target dsDNA, 12.5 nM crRNA, and 0.5 μM 8C-FQ-reporter. The reaction was performed at 37°C in 1× NEB buffer 3.1, and the results were detected in a real-time PCR machine for 30 min with the fluorescence signals collected every 15 s (λex: 488 nm and λem: 535 nm). Finally, the NAD^+^ concentration was calculated according to the standard curve (Y = 388.5X + 36,523) and the dilution factor.

In parallel, cellular NAD^+^ levels were determined by both MS and the NAD^+^/NADH assay kit with MTT (Cominbio and Solarbio), following the manufacturers’ instructions. For the MTT method, cells were washed with cold PBS and were then treated with acidic extraction buffer to release NAD^+^ for subsequent determination by the reduction of MTT to formazan by NADH, which was generated by NAD^+^ reductive reactions with alcohol dehydrogenase.

### 2.7 Cell culture and pharmacological compound treatment

The cell lines of HeLa and 97H were maintained in DMEM/high glucose (HyClone) supplemented with 10% FBS (Gibco) and 1× penicillin–streptomycin (Gibco). The cell lines of HCT116 were maintained in McCoy’s 5A medium (Sigma) supplemented with 10% FBS (Gibco) and 1× penicillin–streptomycin (Gibco). For pharmacological compound treatment, cells at ∼60% confluence were pre-incubated with FK886 (Sigma-Aldrich) at a final concentration of 100 nM and continually cultured for another 24 h before being counted and collected. The cell lines present in this study were obtained from Xiamen Immocell Biotechnology Co., Ltd.

## 3 Results and discussion

### 3.1 Principles of HOLMES(NAD^+^)

**FIGURE 1:**
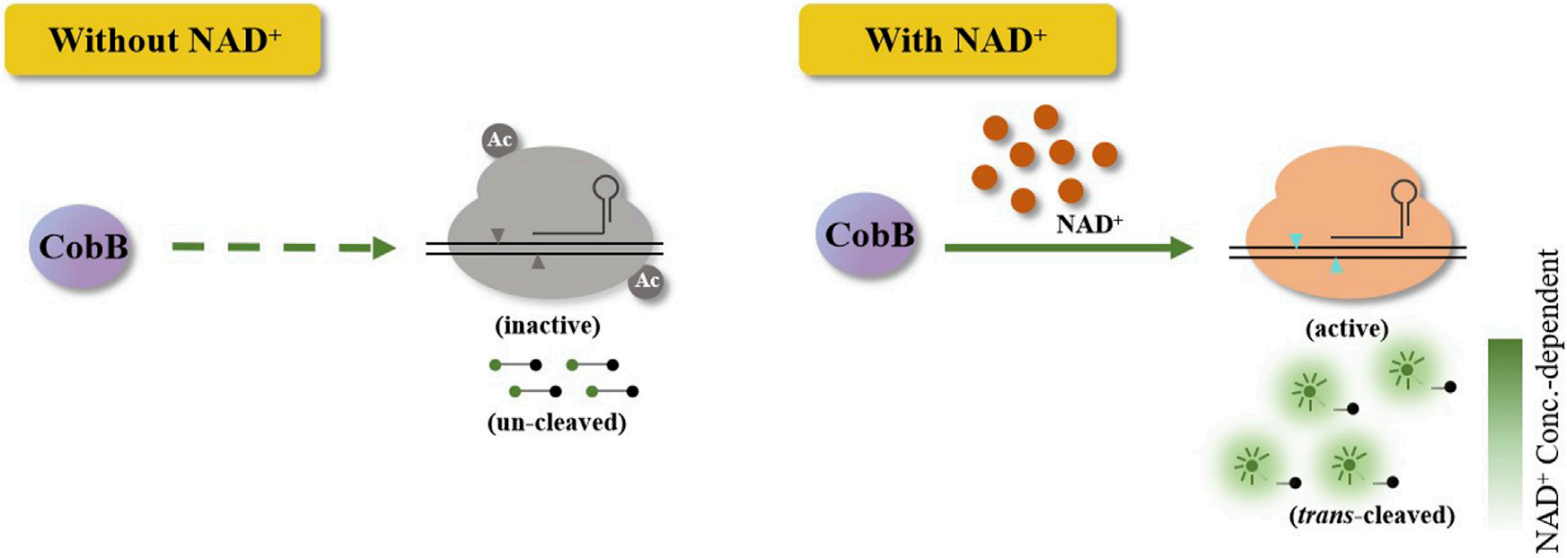
Schematic illustration of the Cas12a-based CRISPR diagnostic system for NAD^+^ detection. Without NAD^+^, acetylated Cas12a loses the *trans*-cleavage activities and is unable to *trans*-cleave ssDNA reporters. When NAD^+^ is added, Cas12a in HOLMES(NAD^+^) is activated, which results in the cleavage of reporters and illumination of fluorescence signals.

Briefly, the HOLMES(NAD^+^) system comprises acetylated Cas12a, CobB, crRNA, target dsDNA, and single-stranded DNA (ssDNA) FQ-reporters. As acetylated Cas12a loses *trans*-cleavage activities with target dsDNA as the template, ssDNA FQ-reporters will not be cleaved by acetylated Cas12a, and no fluorescence signal could be detected. Only in the presence of NAD^+^, CobB then removes the acetyl unit from acetylated Cas12a and restores its *trans*-cleavage activities, cleaving the ssDNA FQ-reporters and illuminating fluorescence signals (Figure 1). Considering the strong relationship between NAD^+^ and the fluorescence signals in HOLMES(NAD^+^), the system can theoretically be employed for NAD^+^ detection.

To test the hypothesis, we first need to produce acetylated Cas12a and establish the HOLMES(NAD^+^) system. Usually, the acetylation process could be performed by the *in vitro* incubation of purified recombinant Cas12a together with AcrVA5, as previously described, where AcrVA5 efficiently acetylates the key lysine residue in Cas12a at the presence of acetyl-CoA (Kang et al., 2022). However, a further step is required to purify the acetylated Cas12a after the *in vitro* reaction, where the procedure is time-consuming and could be harmful to both the activity and stability of Cas12a (Supplementary Figures S1–S3). Therefore, we used an *in vivo* acetylation strategy by purification of acetylated LbCas12a from *E. coli* BL21 (DE3) co-expressing both LbCas12a and AcrVA5. Western blotting results showed that the purified LbCas12a protein was efficiently acetylated (Supplementary Figure S4), and the acetylated LbCas12a (acLbCas12a) lost both *cis-* and *trans-*cleavage activities with target dsDNA (Supplementary Figures S5–S7), which was consistent with our previous study (Kang et al., 2022) and demonstrated the practicability of the *in vivo* acetylation strategy. We further validated that acLbCas12a could be efficiently deacetylated by CobB (Supplementary Figure S8) and recovered its *cis-* and *trans-*cleavage activities when the NAD^+^ cofactor was added. The restored *cis-* and *trans-*cleavage enzymatic activities were comparable to those of the control group using unacetylated Cas12a (Supplementary Figures S5–S7). Notably, the reaction system with acLbCas12a, CobB, crRNA, target dsDNA, and ssDNA FQ-reporters but without NAD^+^ is named the HOLMES(NAD^+^) system.

### 3.2 Performance of HOLMES(NAD^+^)

Then, HOLMES(NAD^+^) stability was analyzed, and no significant difference was found in fluorescence intensities after 20 times of repeated freeze–thaw treatment of the enzyme mixture of Cas12a and CobB (Supplementary Figure S9). When HOLMES(NAD^+^) reactions were tested under temperatures ranging from 30°C to 45°C, the most optimal temperature was determined to be 37°C (Supplementary Figure S10). In addition, we found that HOLMES(NAD^+^) was of both high repeatability (Supplementary Figure S5) and high precision (Supplementary Figure S6). Therefore, the above results clearly showed that HOLMES(NAD^+^) could be triggered by the NAD^+^ cofactor to illuminate fluorescence signals, demonstrating that the system could be employed for stable and accurate NAD^+^ detection.

**FIGURE 2:**
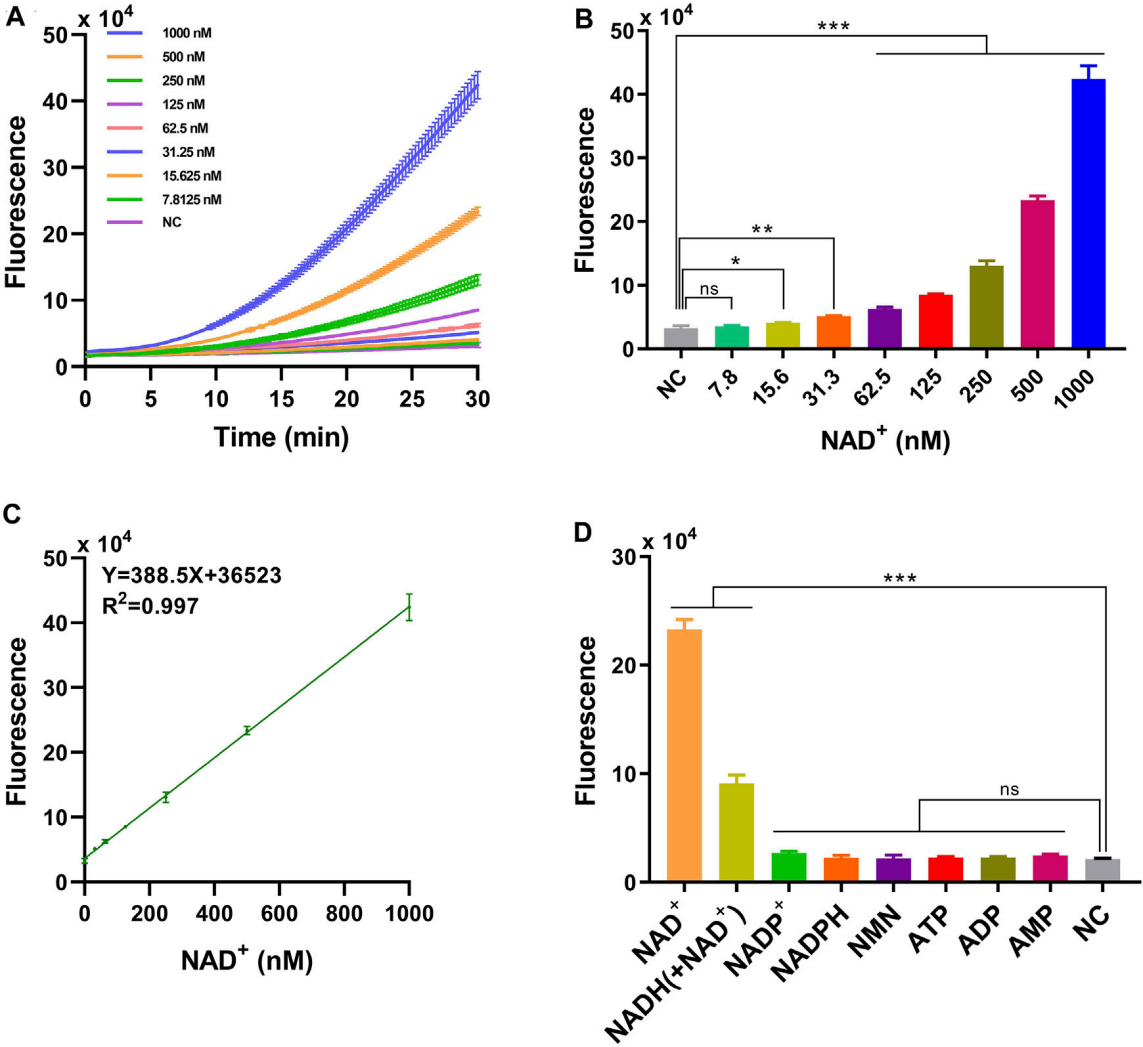
Performance of HOLMES(NAD^+^). **(A)** Time-dependent fluorescence intensities of HOLMES(NAD^+^) in the presence of different concentrations of NAD^+^. **(B)** Fluorescence intensities of HOLMES(NAD^+^) with different concentrations of NAD^+^ added. Concentrations of NAD^+^ ranged from 7.8 nM to 1,000 nM, and the reaction was performed for 30 min (n = 3 technical replicates; two-tailed Student’s *t*-test; *p < 0.1; **p < 0.01; ***p < 0.001; error bars represented the mean with SD). **(C)** Correlation between the fluorescence signals and NAD^+^ concentrations. The solid line represents linear regression fitting to the experimental data. Three replicates were performed for each NAD^+^ concentration. **(D)** Fluorescence intensities of HOLMES(NAD^+^) with various molecules added (n = 3 technical replicates; two-tailed Student’s *t*-test; ***p < 0.001; error bars represented the mean with SD).

The limit of detection (LOD) of HOLMES(NAD^+^) was further determined to be 22.5 nM, using serially diluted NAD^+^ solutions ranging from 1 mM to 7.8 nM. We found that the illuminated fluorescence signals gradually increased with the increase in the NAD^+^ concentration (Figures 2A,B), and the value of the coefficient of determination (*R* ^2^) was 0.997 between the fluorescence intensity and NAD^+^ concentrations (from 1 mM to 31.3 nM) (Figure 2C). The high *R* ^2^ value not only reflected the linear relationship between the added NAD^+^ concentration and the activated LbCas12a *trans*-cleavage activities but also indicated that HOLMES(NAD^+^) could be employed for the quantitative determination of the NAD^+^ concentration.

The specificity of the HOLMES(NAD^+^) system was then tested with the employment of various small molecules, including similar nucleotides and precursors for NAD^+^ biosynthesis. As expected, although some small molecules, such as AMP, ADP, ATP, NMN, NADPH, and NADP^+^ may share similar molecular structures with NAD^+^, they are not the cofactors of CobB and are theoretically unable to restore the *trans*-cleavage activities of acLbCas12a, resulting in no fluorescence signal output. However, to our great surprise, in addition to NAD^+^, HOLMES(NAD^+^) also illuminated fluorescence signals when NADH was supplemented, which is inconsistent with the fact that NAD^+^ is the only cofactor of CobB (Figure 2D). Therefore, we hypothesized that the commercially available NADH was contaminated with NAD^+^, which resulted in the above unexpected signal output for NADH. To test this hypothesis, NADH was then measured by the mass spectrometry (MS) method, and the tested NADH solution was found to be a mixture of NADH and NAD^+^, which could therefore explain the above unexpected results for NADH. Moreover, the concentration of NAD^+^ in the NADH solution (500 nM) was 154.5 nM when measured using the quantitative MS method, which was consistent with the HOLMES(NAD^+^) method, that is, 140.8 nM (Supplementary Figures S16–S19). Taken together, the above findings not only proved the high specificity of the HOLMES(NAD^+^) method but also demonstrated its ability in target NAD^+^ quantitation.

### 3.3 Measurement of NAD^+^ levels in biological samples

**FIGURE 3:**
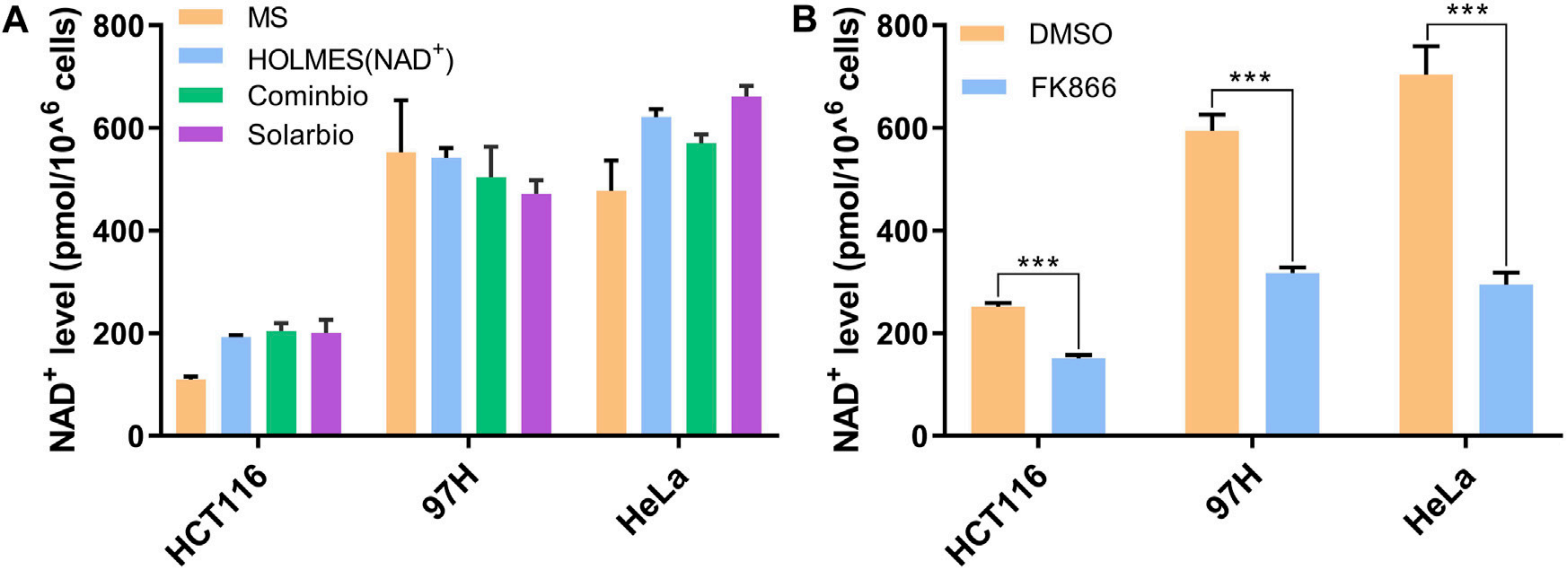
Measurement of NAD^+^ levels in biological samples. **(A)** Quantitative detection of the cellular NAD^+^ levels. Three cancer cell lines of HCT116, 97H, and HeLa were analyzed with three different methods, including the MS method, HOLMES(NAD^+^), and the MTT-based NAD^+^ detection method. Two different commercially available MTT kits from Cominbio and Solarbio were used. **(B)** Quantitative detection of the changes of the cellular NAD^+^ levels after the treatment of FK866 in the cell lines of HCT116, 97H, and HeLa (n = 3 technical replicates; two-tailed Student’s *t*-test; ***p < 0.001; error bars represented the mean with SD).

In addition to pure NAD^+^ solutions, we also verified the HOLMES(NAD^+^) system with extracts from real biology samples. Total NAD^+^ was first extracted from different cancer cell lines by heating at 95°C and then neutralized before being detected. Using the HOLMES(NAD^+^) method, the cellular NAD^+^ levels were identified to be approximately 200–600 pmol cells. The samples were also measured with other methods in parallel, and the results were consistent with those of the MS and MTT methods (Figure 3A). Moreover, the HOLMES(NAD^+^) method was further used to quantitatively measure the changes in the cellular NAD^+^ concentrations, including 97H, HeLa, and HCT116. After being treated with FK866, an inhibitor of the nicotinamide phosphoribosyltransferase, the cellular NAD^+^ levels dropped in all tested cell lines (Figure 3B), which is consistent with the previous report (Pittelli et al., 2010).

Previous studies have uncovered the potential of CRISPR technologies in non-nucleic acid detection, and the targets include both proteins (e.g., microcystin-LR, thrombin, and IFN-γ) (Deng et al., 2021; Wang et al., 2021; Wu et al., 2021) and small molecules (e.g., ATP, antibiotics, uric acid, and melamine) (Liang et al., 2019; Qiao et al., 2021; Chen et al., 2022; Mahas et al., 2022). Here, with the use of NAD^+^-based posttranslational modification of Cas12a, we create an NAD^+^-specific CRISPR diagnostic system, namely, HOLMES(NAD^+^), further expanding the Cas12a application in non-nucleic acid detection scenarios. Through comparison of different NAD^+^ detection methods, we find that different methods show distinct advantages and disadvantages (Supplementary Table S7). For example, although the MS method requires expensive instruments and professional operations, it can measure more than one target molecules in one test and allow for organic extraction that may better preserve molecules, such as NADH and NADPH (Yamada et al., 2006). HOLMES(NAD^+^) and other methods, by contrast, detect merely the target NAD^+^ concentration, which could be less convenient when the detection of multiple molecules is needed. In addition, we also observed that the detection efficiencies of the enzyme-based HOLMES(NAD^+^) and MTT methods might be slightly affected by intracellular substances, i.e., the efficiencies are enhanced in the HCT116 and HeLa samples, but reduced in the 97H sample in comparison with the MS method. Overall, HOLMES(NAD^+^) shows apparent advantages in rapidness (half an hour), convenience, cost-effectiveness, and accuracy in the quantitative detection of NAD^+^ concentrations, which therefore shows great potential in both scientific research and industries.

## Data Availability

The original contributions presented in the study are included in the article/Supplementary Material; further inquiries can be directed to the corresponding authors.
