# Supplementary material for: CRISPR-HOLMES-based NAD+ detection
Source: Front Bioeng Biotechnol. 2024 Mar 25;12:1355640. doi: 10.3389/fbioe.2024.1355640 (PMC10999544; doi:10.3389/fbioe.2024.1355640)
Supplement: Supplementary file 1 [file DataSheet1.docx]

CRISPR-HOLMES-based NAD^+^ detection

Supplementary Material

# Supplementary Tables

**Table S1. Oligonucleotides used for plasmids construction in this study.**

| **Oligo names** | **Sequences (5'-3')** |
| --- | --- |
| LbCas12a-F | cgcGGATCCcatgctgaagaacgtgggcatcga |
| LbCas12a-R | cgcGTCGACtcagtgtttcacgctggtctgagcata |

**Table S2. Oligonucleotides used for preparation of LbCas12a targets in this study.**

| **Oligo names** | **Sequences (5'-3')** |
| --- | --- |
| T1-50 nt-F | gttgtaaaacgacggccagttttgttatcgcaactttctactgaattcgg |
| T1-50 nt-R | ccgaattcagtagaaagttgcgataacaaaactggccgtcgttttacaac |
| M13-F | TGTAAAACGACGGCCAGT |
| M13-R-FAM | FAM-CAGGAAACAGCTATGACC |

**Table S3. The crRNA and FQ-reporter used in this study.**

| **Oligo names** | **Sequences (5'-3')** |
| --- | --- |
| crRNA | AAUUUCUACUCUUGUAGAUUUAUCGCAACUUUCUACUGAAUU |
| FQ-reporter | 5’-FAM-CCCCCCCC-BHQ1-3’ |

**Table S4. Similar nucleotides and biosynthetic precursors for NAD^+^.**

| **Name** | **Provider** | **Cat. Nos.** |
| --- | --- | --- |
| NAD^+^ | Sigma | N7004-250MG |
| NADH | MCE | HY-F0001 |
| NADP^+^ | MERCK | 24292-60-2 |
| NADPH | Sigma | N1630-250MG |
| NMN | RHAWN | R019041-100MG |
| ATP | Sigma | A6419-1G |
| ADP | YONGQI | 20398-34-9 |
| AMP | YONGQI | 4578-31-8 |

**Table S5. Repeatability test of HOLMES(NAD^+^) detection methods.**

| **NAD^+^ conc. (nM)** | **1**  **(Fluorescence)** | **2**  **(Fluorescence)** | **3**  **(Fluorescence)** | **Mean**  **(Fluorescence)** | **RSD**  **(%)** |
| --- | --- | --- | --- | --- | --- |
| 1,000 | 430406.063 | 464732.469 | 449093.844 | 448077.458 | 3.84 |
| 600 | 277618.563 | 257968.734 | 263600.938 | 266396.078 | 3.80 |
| 150 | 85089.156 | 95409.117 | 92321.094 | 90939.789 | 5.82 |

**Table S6. Precision test of HOLMES(NAD^+^) detection methods.**

| **NAD^+^ conc. (nM)** | **1**  **(nM)** | **2**  **(nM)** | **3**  **(nM)** | **4**  **(nM)** | **5**  **(nM)** | **Mean**  **(nM)** | **RSD**  **(%)** | **Measured**  **/True values** |
| --- | --- | --- | --- | --- | --- | --- | --- | --- |
| 1,000 | 1055.263 | 1070.972 | 1103.856 | 1083.544 | 1081.335 | 1078.994 | 1.65 | 1.079 |
| 500 | 517.028 | 489.253 | 532.828 | 509.723 | 495.917 | 508.950 | 3.39 | 1.018 |
| 100 | 103.955 | 80.211 | 89.669 | 93.992 | 99.005 | 93.367 | 8.72 | 0.934 |

**Table S7. Comparison of different NAD^+^ detection methods.**

|  | Sensitivity | Specificity | Accurate | Labor  intensive | Expensive equipment | Time/min | References |
| --- | --- | --- | --- | --- | --- | --- | --- |
| Enzymatic assay | 8.3 nM | YES | YES | YES | NO | 50 | (Bernofsky and Swan, 1973) |
| HPLC-UV | 1 μM | YES | YES | YES | YES | 40 | (Jones, 1981) |
| LC/MS/MS | 10 nM | YES | YES | YES | YES | 35 | (Yamada et al., 2006) |
| NAD-CPLuc^2^ | 20 nM | NO | YES | NO | NO | 10 | (Yu et al., 2019) |
| HOLMES(NAD**^+^**) | 22.5 nM | YES | YES | NO | NO | 30 | This work |

**Supplementary Figures**

**
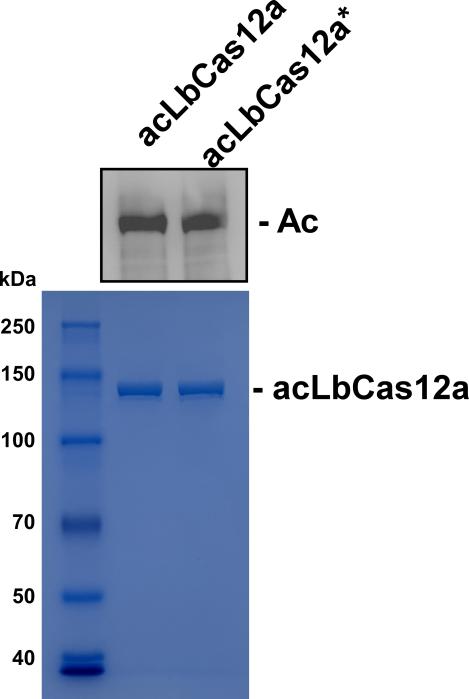
**

**Supplementary Figure 1. Western blotting analysis of the lysine acetylation levels using acLbCas12a produced by both *in vitro* and *in vivo* approaches.** The acLbCas12a proteins were obtained from *E. coli* co-expressing both LbCas12a and AcrVA5, designated as the *in vivo* method. The acLbCas12a* proteins were obtained by AcrVA5-mediated *in vitro* acetylation treatment of LbCas12a protein.

**
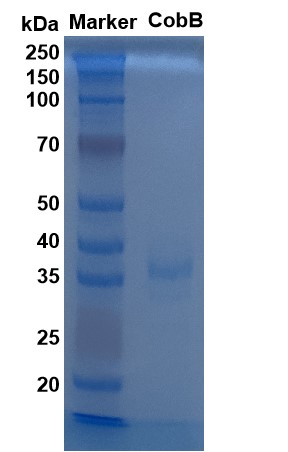
**

**Supplementary Figure 2. SDS-PAGE analysis of purified recombinant CobB protein.**

**
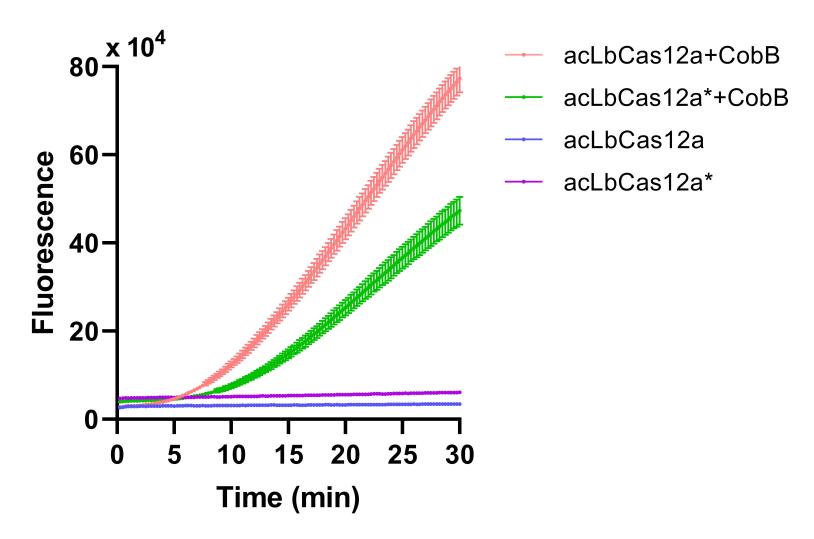
**

**Supplementary Figure 3. Time-dependent fluorescence intensities of the Cas12a *trans*-cleavage experiments.** Assays were performed with different acLbCas12a obtained from either *in vivo* or *in vitro* (*) methods. acLbCas12a was treated with or without the addition of CobB deacetylation components.

**
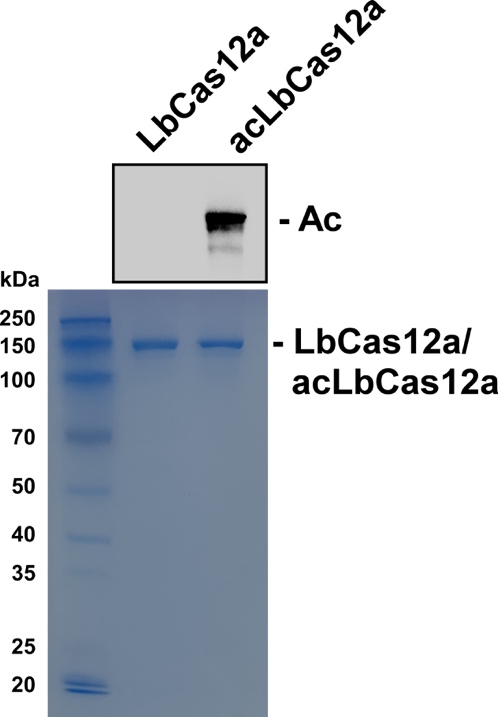
**

**Supplementary Figure 4.** **Western blotting analysis of the acetylated LbCas12a (acLbCas12a).** Both LbCas12a and acLbCas12a were analyzed by SDS-PAGE, followed by WB analysis.

**
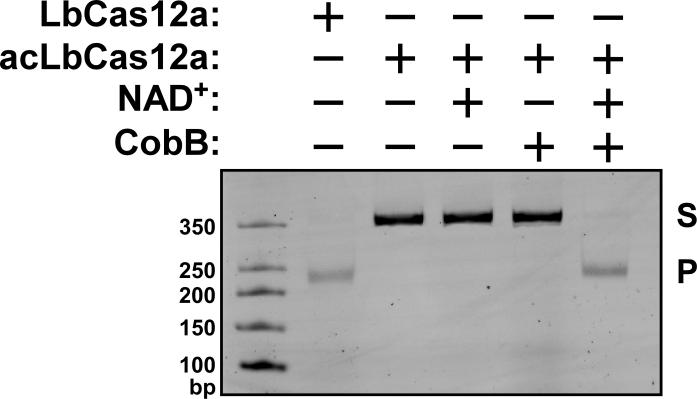
**

**Supplementary Figure 5. *Cis*-cleavage assay with acLbCas12a.** acLbCas12a was treated with different conditions with or without CobB and NAD^+^. S, dsDNA substrate; P, Cas12a *cis*-cleaved products.

**
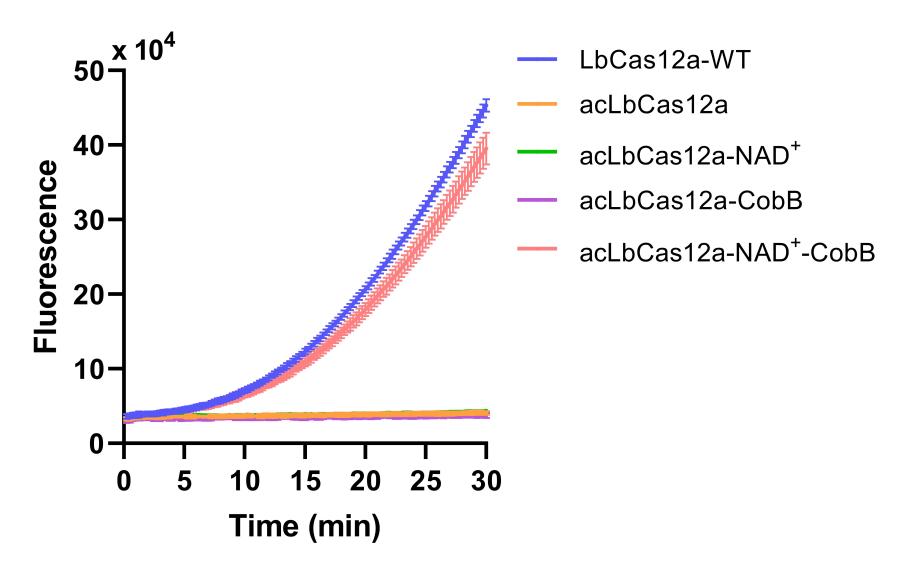
**

**Supplementary Figure 6. Time-dependent fluorescence intensities of the acLbCas12a *trans*-cleavage experiment.** acLbCas12a was treated in conditions with or without the addition of CobB and NAD^+^.

**
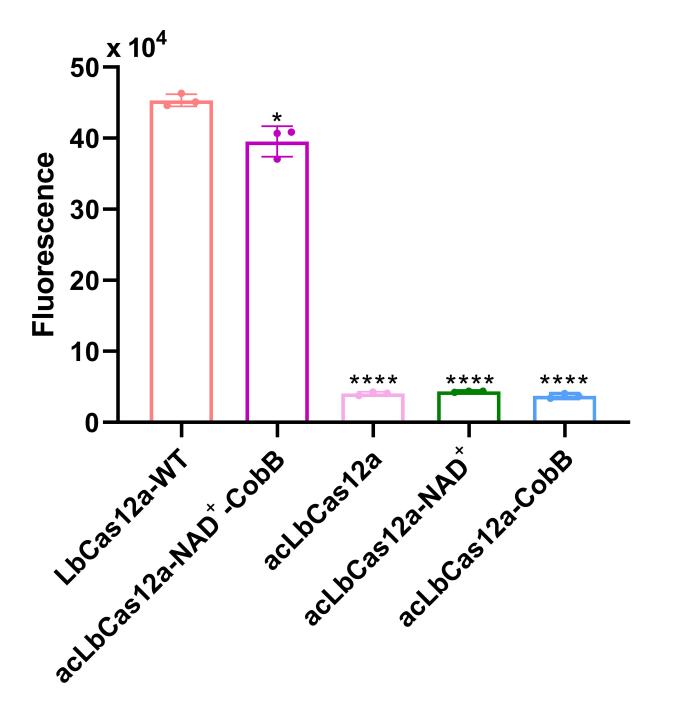
**

**Supplementary Figure 7. *Trans*-cleavage assay with acLbCas12a.** Assay was performed with acLbCas12a treated with or without the addition of CobB and NAD^+^. The presented fluorescence intensities were obtained by incubating the reaction at 37 ℃ for 30 min (n=3 technical replicates; two tailed Student’s t-test; * p<0.1; **** p<0.0001; error bars represent the mean with SD).

**
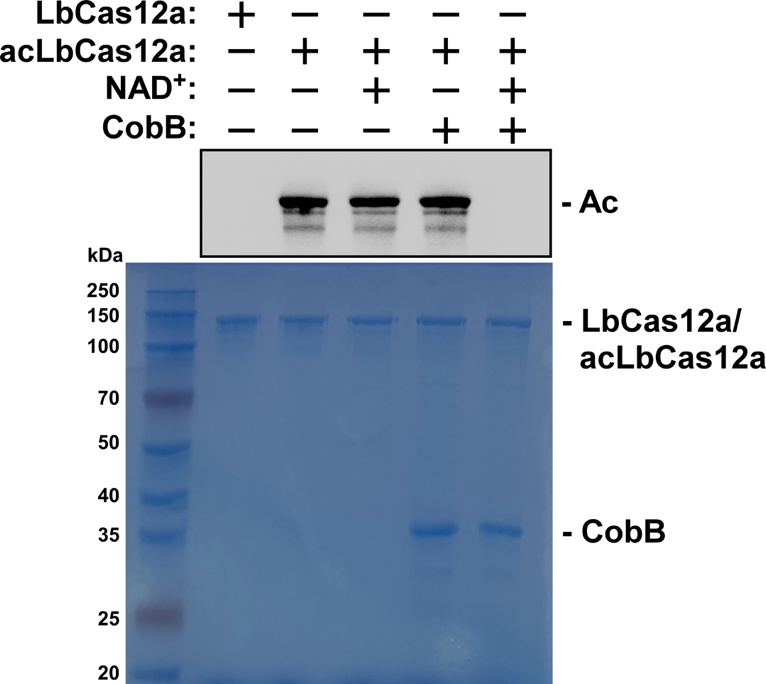
**

**Supplementary Figure 8. WB analysis of the acetylation status of acLbCas12a.** acLbCas12a was treated by CobB-mediated deacetylation in conditions with or without the addition of CobB and NAD^+^. α-Kac, pan anti-acetyl lysine antibody (PTM Bio, Cat# PTM-001).


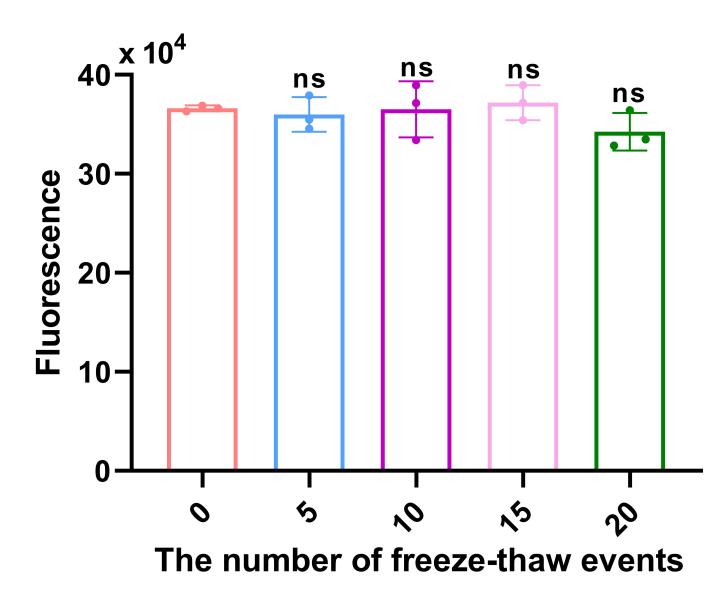


**Supplementary Figure 9. The effect of freeze-thaw treatment on the stability of the proteins in HOLMES(NAD^+^) system.** The acLbCas12a/CobB protein mixture was first frozen at -80 ℃ and then thawed at 37 ℃. After repeated freeze-thaw treatment, the protein mixture was used for HOLMES(NAD**^+^**) analysis. The fluorescence intensities were obtained through incubating the reaction at 37 ℃ for 30 min (n=3 technical replicates; two tailed Student’s t-test; error bars represent the mean with SD).


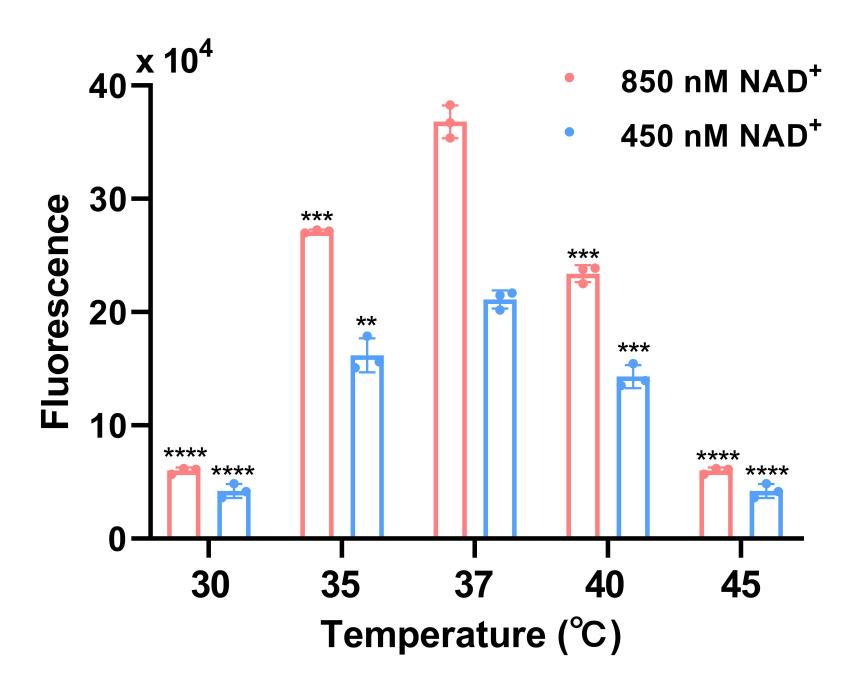


**Supplementary Figure 10. Analysis of the applicable temperature range for HOLMES(NAD^+^).** HOLMES(NAD**^+^**) reaction was performed at designated temperatures using two different NAD^+^ concentrations of both 850 nM and 450 nM. The fluorescence intensities were obtained through incubating the reaction for 30 min (n=3 technical replicates; two tailed Student’s t-test; ** p<0.01; *** p<0.001; **** p<0.0001; error bars represent the mean with SD).


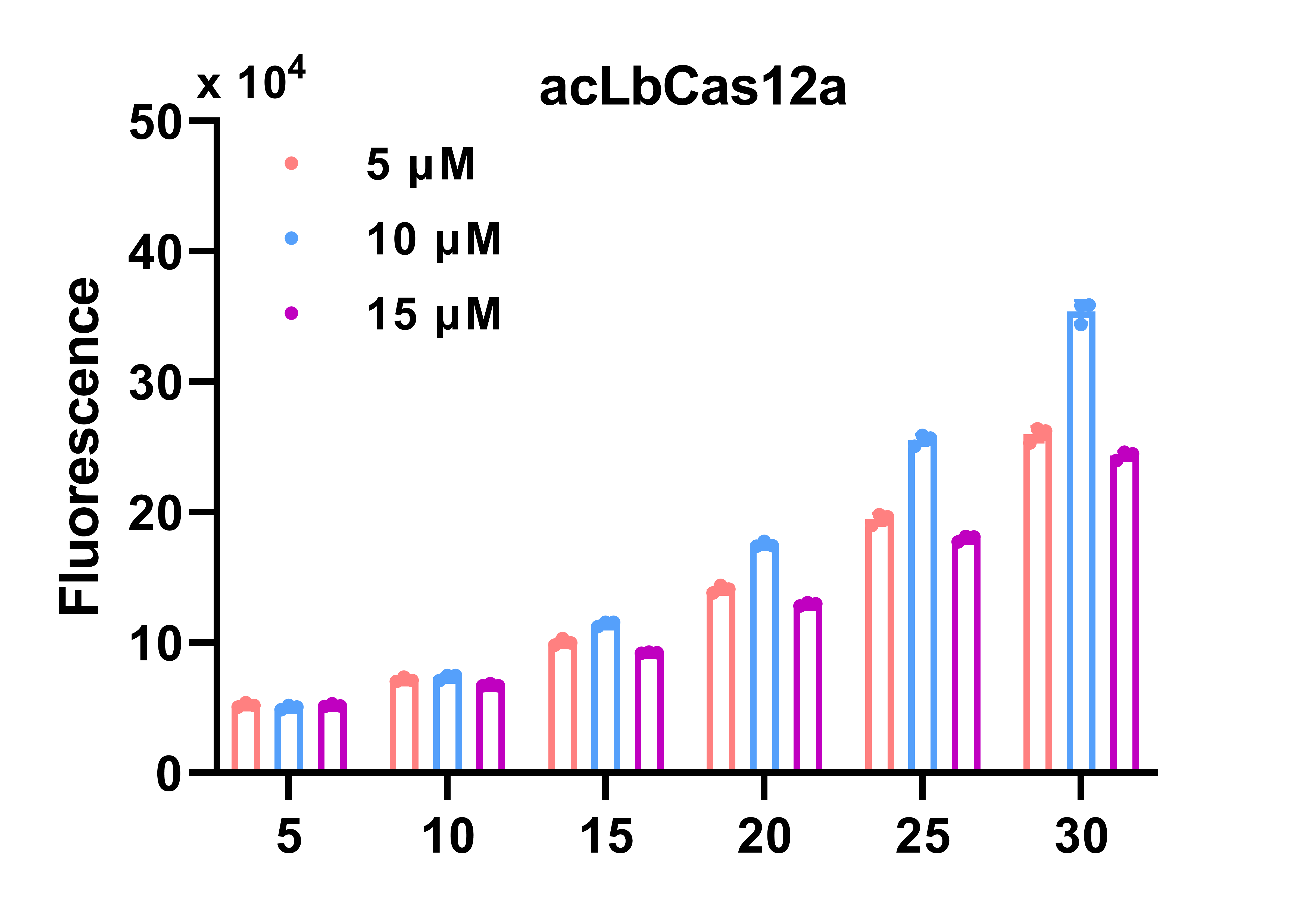


**Supplementary Figure 11. Optimization of the range of acLbCas12a concentrations for HOLMES (NAD^+^).** HOLMES(NAD**^+^**) reaction was performed at designated concentrations using three different acLbcas12a concentrations of 5 μM, 10 μM and 15 μM. The fluorescence intensities were obtained through incubating the reaction at 37 ℃ for 30 min (n=3 technical replicates; two tailed Student’s t-test; error bars represent the mean with SD).


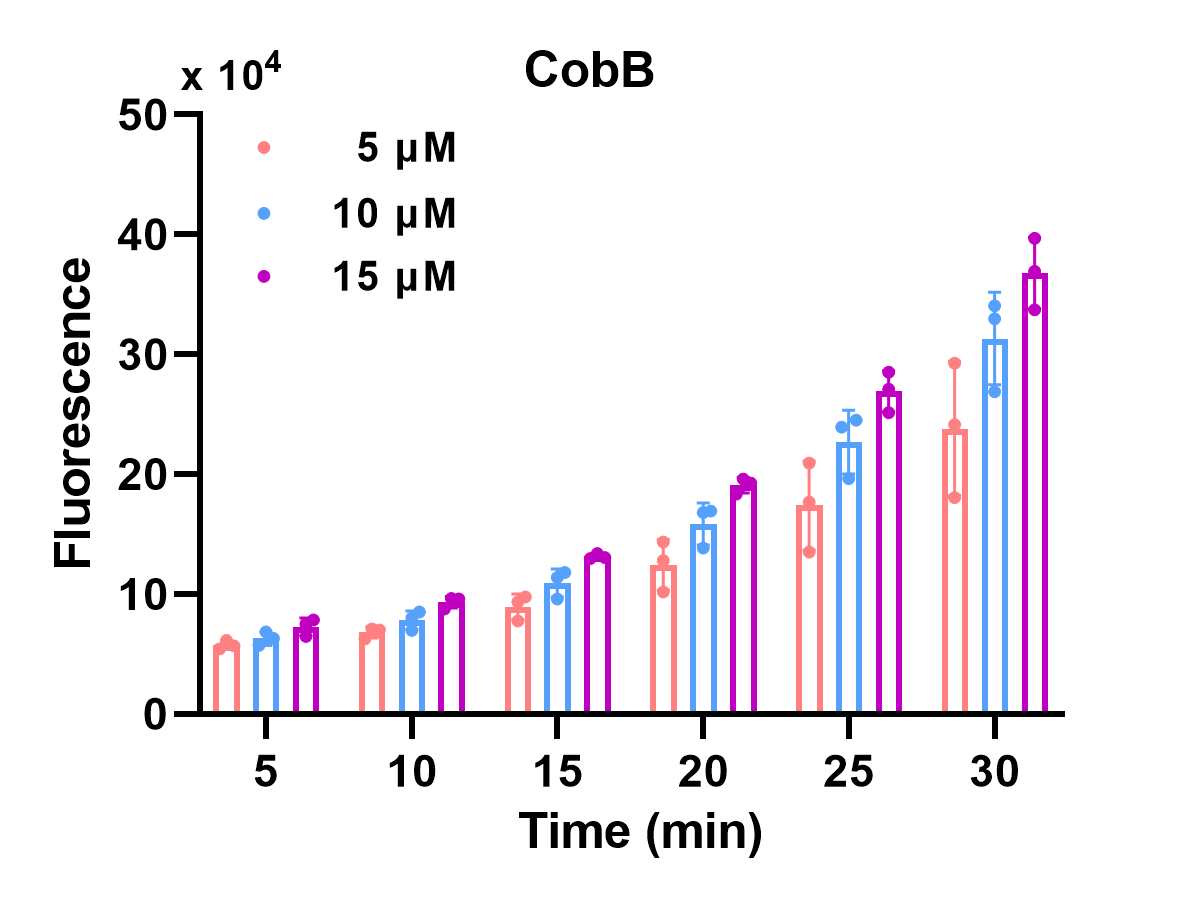


**Supplementary Figure 12. Optimization of the range of CobB concentrations for HOLMES (NAD^+^).** HOLMES(NAD**^+^**) reaction was performed at designated concentrations using three different CobB concentrations of 5 μM, 10 μM and 15 μM. The fluorescence intensities were obtained through incubating the reaction at 37 ℃ for 30 min (n=3 technical replicates; two tailed Student’s t-test; error bars represent the mean with SD).

**
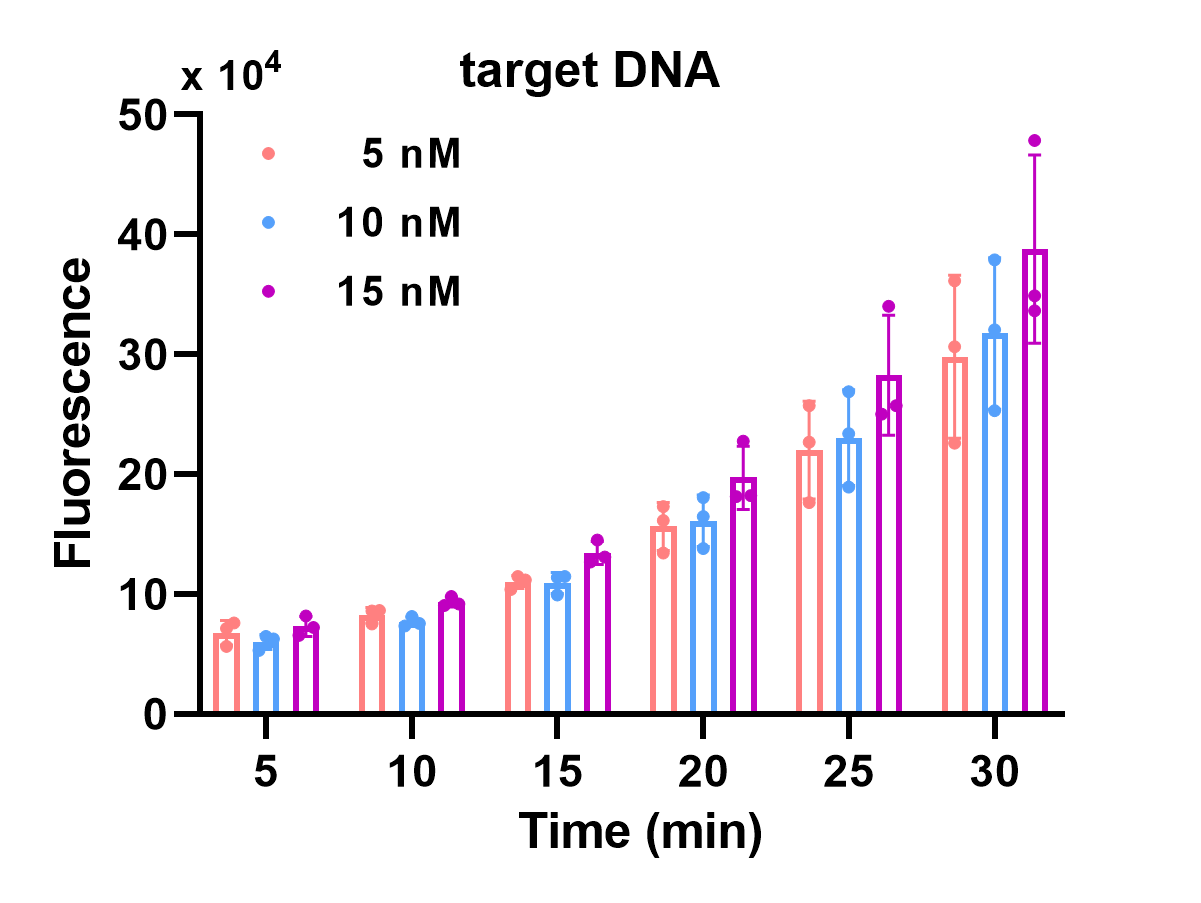
**

**Supplementary Figure 13. Optimization of the range of target DNA concentrations for HOLMES (NAD^+^).** HOLMES(NAD**^+^**) reaction was performed at designated concentrations using three different target DNA concentrations of 5 μM, 10 μM and 15 μM. The fluorescence intensities were obtained through incubating the reaction at 37 ℃ for 30 min (n=3 technical replicates; two tailed Student’s t-test; error bars represent the mean with SD).

**
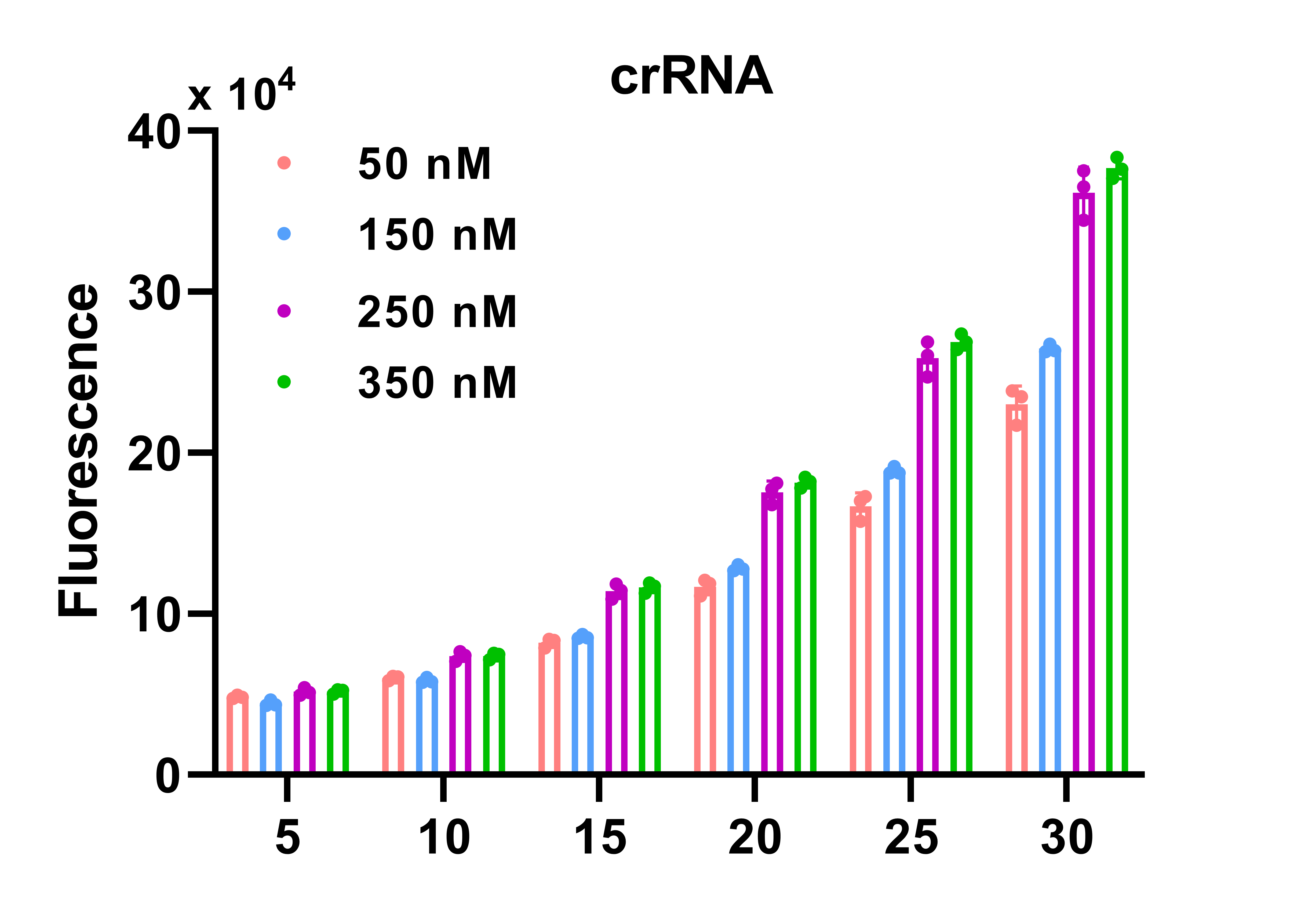
**

**Supplementary Figure 14. Optimization of the range of crRNA concentrations for HOLMES (NAD^+^).** HOLMES(NAD^+^) reaction was performed at designated concentrations using four different target DNA concentrations of 50 nM, 150 nM, 250 nM and 350 nM. The fluorescence intensities were obtained through incubating the reaction at 37 ℃ for 30 min (n=3 technical replicates; two tailed Student’s t-test; error bars represent the mean with SD).

**
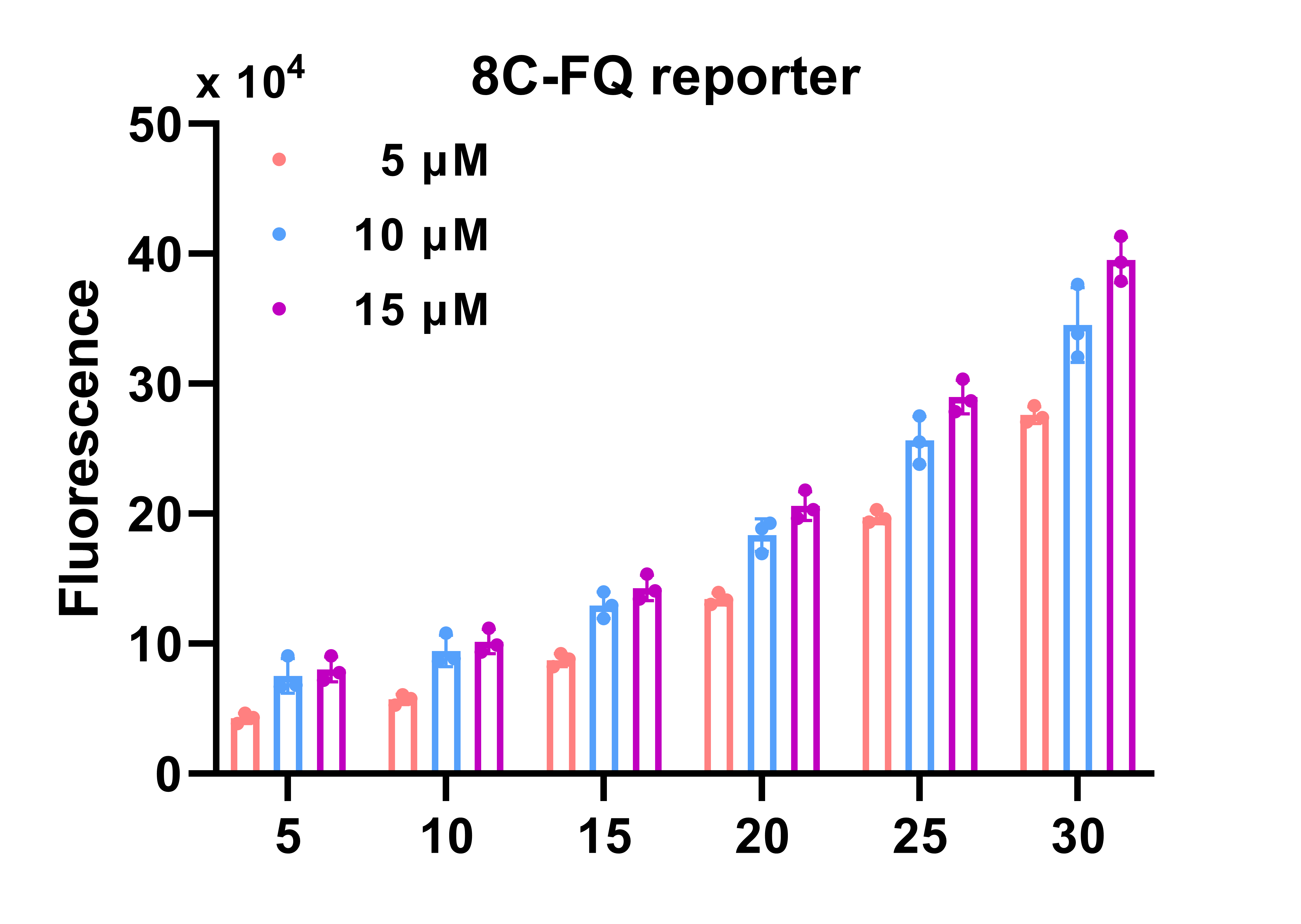
**

**Supplementary Figure 15. Optimization of the range of 8C-FQ reporter concentrations for HOLMES (NAD^+^).** HOLMES(NAD^+^) reaction was performed at designated concentrations using three different 8C-FQ reporter concentrations of 5 μM, 10 μM and 15 μM. The fluorescence intensities were obtained through incubating the reaction at 37 ℃ for 30 min (n=3 technical replicates; two tailed Student’s t-test; error bars represent the mean with SD).


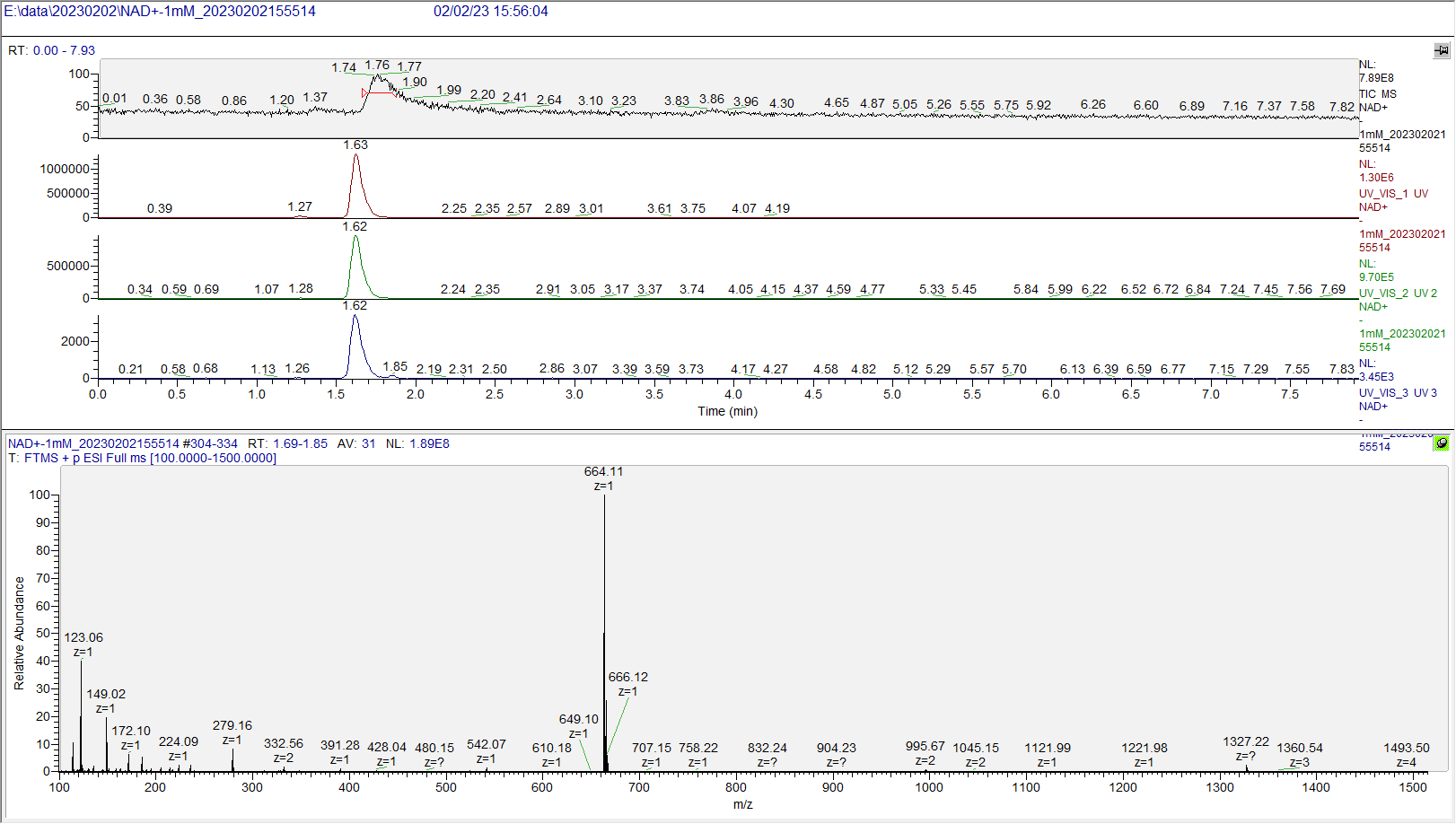

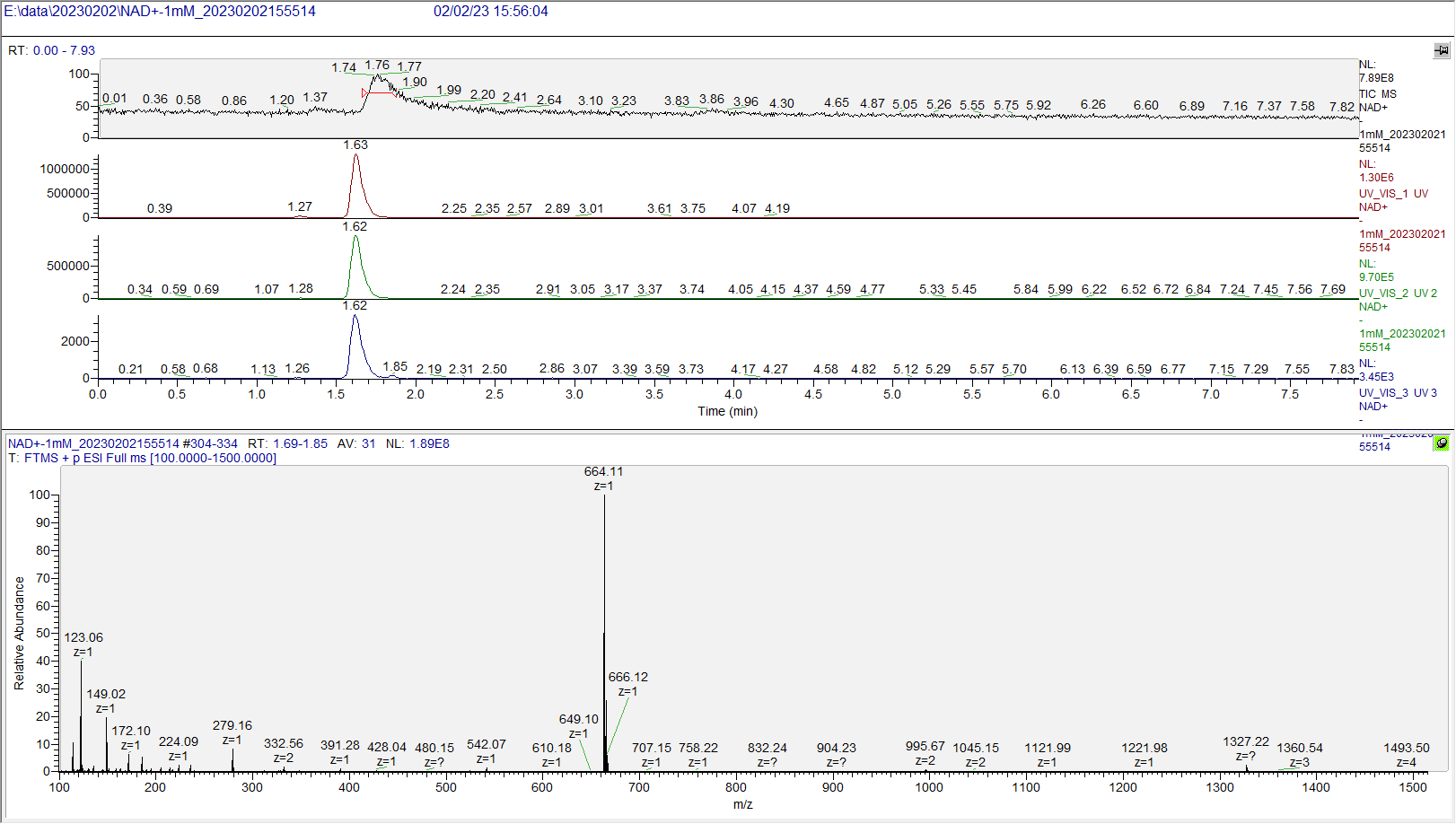


**Supplementary Figure 16.** **Analysis of the composition of the NAD^+^ standards with mass spectrometry (MS).** Main component of the MS signals collected at retention time from 1.69 to 1.85 minutes was NAD^+^.


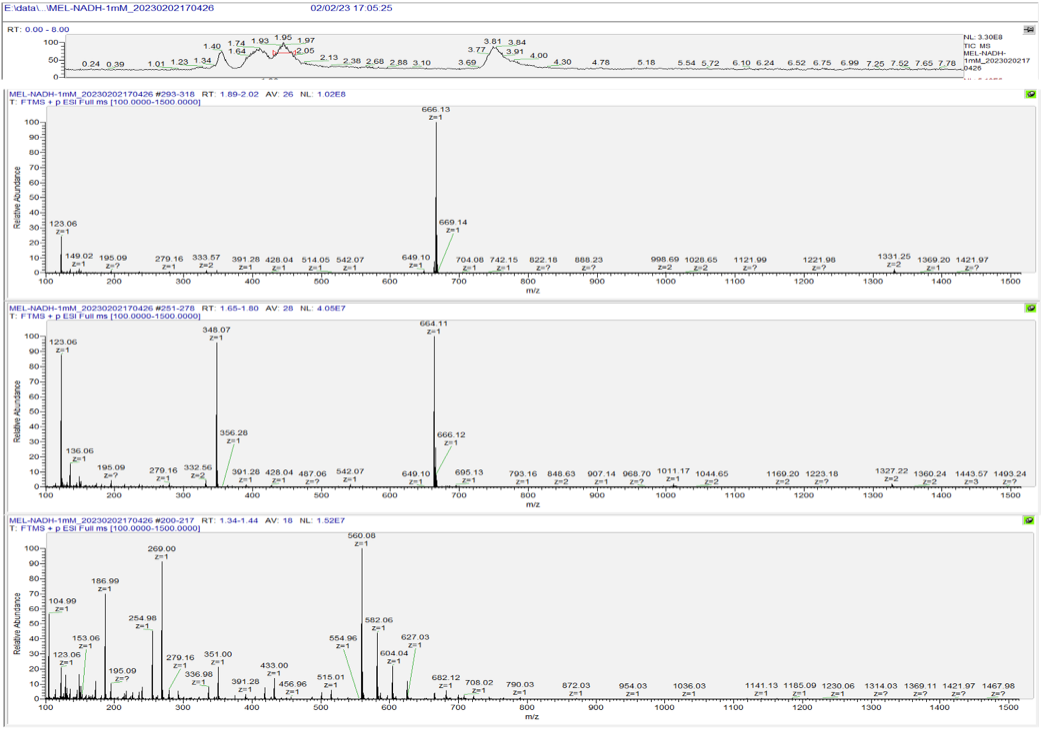


**Supplementary Figure 17. Analysis of the composition of the NADH standards with mass spectrometry (MS).** Main component of the MS signals collected at retention time from 1.89 to 2.02 minutes was NADH, while the component from 1.65 to 1.80 minutes was NAD^+^. Components with retention time from 1.34 to 1.44 minutes were contaminants.


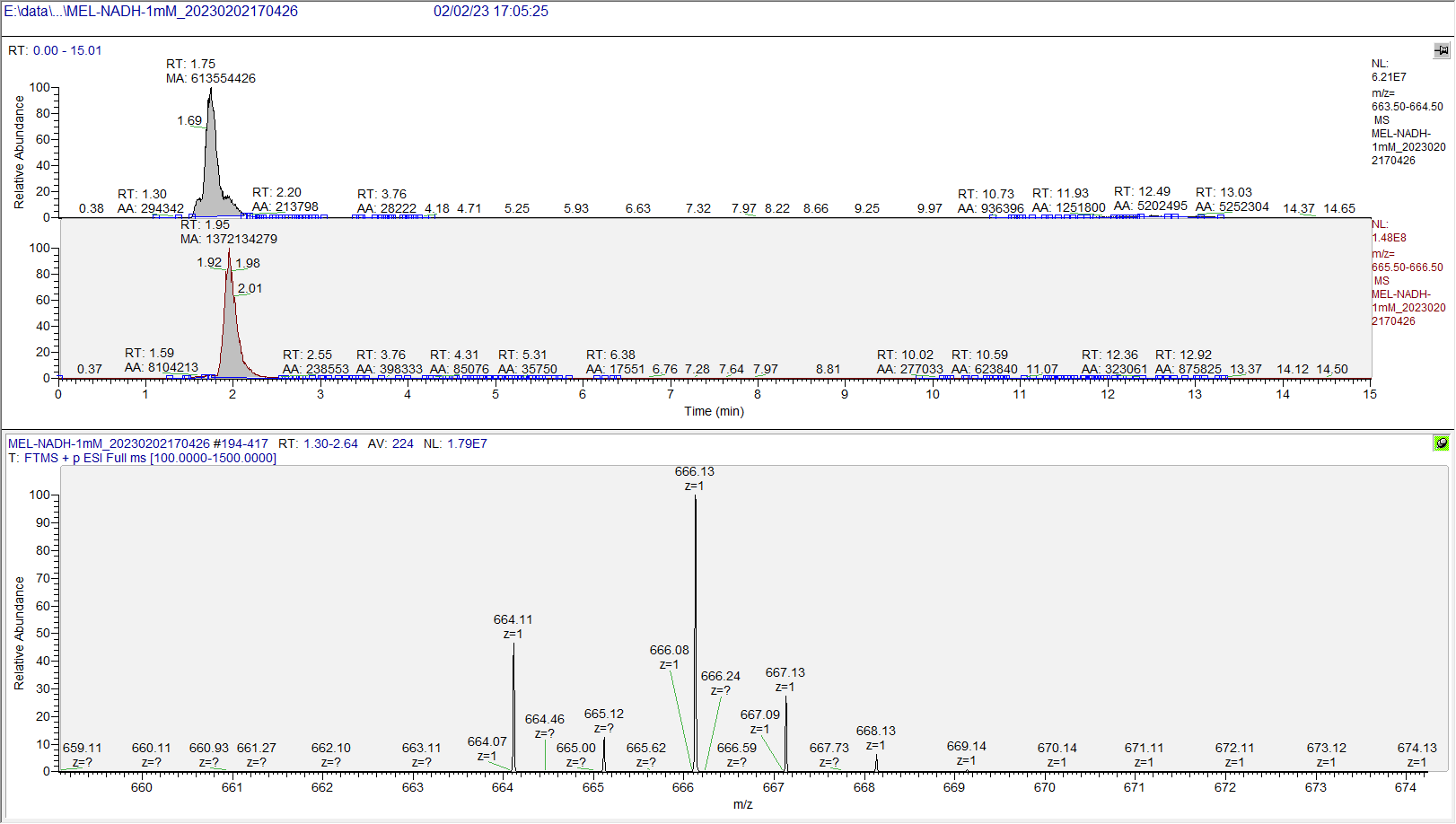


**Supplementary Figure 18. Quantitative analysis of the NAD^+^ and NADH contents in the NADH standards by mass spectrometry.** The NAD^+^ peak area was 613,554,426, and the NADH peak area was 1,372,134,279.

**
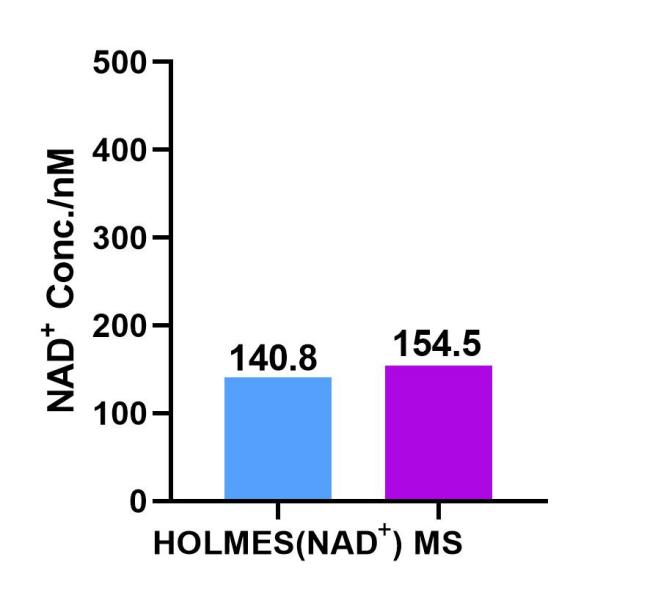
**

**Supplementary Figure 19. Calculation of the NAD^+^ contents in the NADH standards.** The contents were analyzed with both the HOLMES(NAD^+^) method and the MS method. For the HOLMES(NAD^+^) method, fluorescence intensities of the NADH standard were converted to the NAD^+^ concentration using the formula from the standard curve, which is Y= 388.5X +36523. While for the MS method, the NAD^+^ content was determined using the following formula the concentration of [NADH]*(peak areas [NAD^+^]/peak areas ([NAD^+^]+[NADH])).

**References**

Bernofsky, C., and Swan, M. (1973). An improved cycling assay for nicotinamide adenine dinucleotide. *Analytical Biochemistry* 53**,** 452-458.

Jones, D.P. (1981). Determination of pyridine dinucleotides in cell extracts by high-performance liquid chromatography. *Journal of Chromatography B Biomedical Sciences & Applications* 225**,** 446-449.

Yamada, K., Hara, N., Shibata, T., Osago, H., and Tsuchiya, M. (2006). The simultaneous measurement of nicotinamide adenine dinucleotide and related compounds by liquid chromatography/electrospray ionization tandem mass spectrometry. *Analytical Biochemistry* 352**,** 282-285.

Yu, Q., Pourmandi, N., Xue, L., Gondrand, C., and Johnsson, K. (2019). A biosensor for measuring NAD+ levels at the point of care. *Nature Metabolism* 1**,** 1219-1225.
